# Implementing Arts on Prescription at Home for People Living With Dementia: A Hybrid-Effectiveness Feasibility Study

**DOI:** 10.1177/08919887241267335

**Published:** 2024-07-23

**Authors:** Claire MC O'Connor, Roslyn G Poulos, Michelle Heldon, Costanza Preti, Elizabeth Beattie, Christopher J Poulos

**Affiliations:** 1 School of Psychology, 7800 University of New South Wales, Sydney, NSW, Australia; 2 Centre for Positive Ageing, 94268 HammondCare, Sydney, NSW, Australia; 3 Neuroscience Research Australia, Sydney, NSW, Australia; 4 Ageing Futures Institute, 567274 University of New South Wales, Sydney, NSW, Australia; 5 School of Population Health, 7800 University of New South Wales, Sydney, NSW, Australia; 6 1969 School of Nursing, Queensland University of Technology, Brisbane, QLD, Australia

**Keywords:** arts programs, dementia, service development, implementation, rehabilitation

## Abstract

Arts on prescription at home (AoP@Home) is a participatory art-based approach involving a professional artist engaging a person with dementia (and their family carer) in art-making in their own home. This study evaluated the implementation of AoP@Home within a real-world community aged care context. A hybrid effectiveness-implementation design was used to simultaneously test both the AoP@Home intervention and the implementation process. AoP@Home program outcomes included person with dementia and family carer (dyad) health and wellbeing, and personal goal attainment. Implementation outcomes were evaluated according to feasibility, fidelity, acceptability, uptake, and costs via routinely collected data, artist notes, and interviews with program managers, artists, and participant dyads. Four dyads completed an AoP@Home program during the study period. All participants with dementia reported improvements in their overall health and wellbeing, and wellbeing scores improved for all carers from baseline to post-program. Implementation was feasible using existing government funding mechanisms, and programs were acceptable to all stakeholders. It is possible to deliver participatory arts programs for community-dwelling people with dementia and their family, in their home, using sustainable and available funding models. Programs such as AoP@Home should be made more accessible alongside broader allied health and care services.

## Introduction

Dementia is a progressive and (as yet) incurable condition that leads to functional decline and can result in changes in memory, cognition, communication, personality, behaviour, social ability, physical ability, and self-care.^1^ While it is more common in older people, dementia is not a normal part of the ageing process, and indeed, can impact people of all ages. The recently released World Health Organisation Package of Rehabilitation Interventions recognises the disabling impact of dementia, and how this extends beyond the person living with dementia to also include their family and broader society, with great economic implications.^2^ The lives of people with dementia and their carers can be markedly improved with early recognition, supportive treatment and rehabilitation to optimise function and well-being.^3^

The importance of the arts in supporting health and wellbeing is increasingly recognised.^4,5^ For people with dementia, participating in arts-based activities and interventions has been recognised as having potential to impact on wellbeing and quality of life,^6^ with activities such as making music, visual arts, and dance found to positively impact cognitive and social functioning.^7^ Arts on prescription (AoP) is a participatory art-based approach that has shown promise for people with dementia in both group-based and in-home settings.^5,8,9^ Participatory art is different from art therapy as it places a larger emphasis on engagement in the art-making process, rather than on psychological outcomes.^10^ Participatory art, as in AoP, is a form of social prescribing, which is where health professionals refer their clients to activities that are delivered in the community.^11–13^ AoP sessions are run by artists, and can involve a range of art and music modalities ^12^ with the aim of improving mental health and wellbeing.^14^

Participatory arts are ‘prescribed’ for AoP participants to emphasise the importance of this approach as a legitimate option alongside other allied health reablement interventions.^5,15^ In its original form, AoP involves groups of six to eight clients being actively engaged in art-making with a professional artist. While benefits of this group-based format have been identified for older people, including people with dementia,^5,16^ accessing community-based activities (often) becomes increasingly difficult for people as their dementia progresses.^17^ To address this challenge around program access, a model of AoP at home (AoP@Home) was devised, where the professional artist delivers eight to 10 participatory art sessions in the client’s home.^8,18^ AoP@Home is a dyadic program, where both the person with dementia and their family carer(s) are invited to participate. A recent pilot of AoP@Home identified positive outcomes for both members of the dyad after engaging in the program, with specific benefits including gaining a sense of achievement, improved social interactions, and having experienced ‘flow’ during art sessions where everyday worries were muted by absorption in the art-making process.^8^

While the AoP@Home pilot study^8^ provided initial evidence for positive benefits of the program, an important piece of the puzzle remained unaddressed. There remains a lack of research into how to implement AoP@Home within a real-world community aged care context, using existing available funding models. A vital preliminary step towards this process was recently undertaken in a study that identified the barriers and enablers to implementing AoP@Home within a community aged care context.^19^ The barriers and enablers identified were centred around four key themes: awareness and engagement in the sector (i.e. the need for key stakeholders in the community aged care sector to gain more knowledge of AoP@Home), artists delivering programs (i.e. developing skills to work effectively with people with dementia), awareness and engagement of people impacted by dementia (i.e. supporting program uptake and engagement through tailored information and open communication), and practicalities of implementation (i.e. considerations around staffing, referrals, funding etc). The outcomes from this preliminary study were used to underpin the implementation strategy applied in the current study.^19^

This study evaluated the implementation of AoP@Home within a real-world community aged care context. Implementation research is a vital step towards the effective translation of research evidence into healthcare practice and policy.^20^ As with intervention trials, pilot and feasibility implementation studies facilitate testing of implementation strategies and identification of any need for strategy refinement prior to a larger implementation trial.^21^ In addition to evaluating the implementation process, an important aspect of this study was to build on the preliminary positive evidence for AoP@Home program outcomes for people with dementia and their family carers. Research into socially prescribed activities has identified an important need to evaluate programs in order to monitor program effectiveness and provide feedback to referring health practitioners.^14,22^ A hybrid effectiveness-implementation design was used to facilitate this dual feasibility testing of both the AoP@Home intervention and the implementation strategies.^23^ As such, the aims of this study were twofold: (1) to evaluate feasibility of measuring AoP@Home program outcomes for people living with dementia and their family carers, and (2) to evaluate the implementation of AoP@Home within an existing community aged care service.

## Materials and methods

### Study Design and Setting

**Figure 1.**
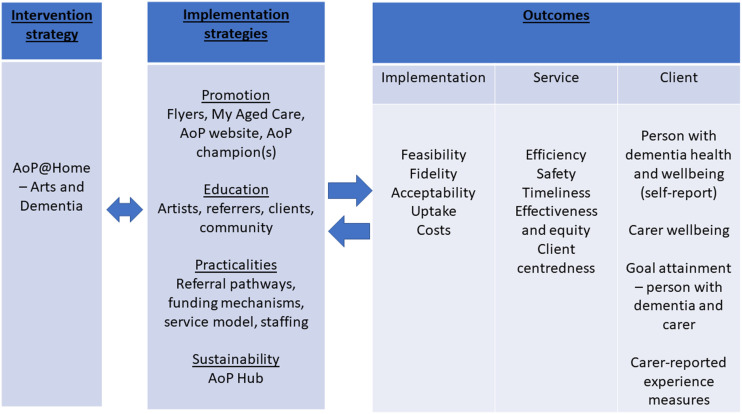
Hybrid effectiveness-implementation evaluation framework. *Notes*: Adapted from Proctor et al. 2011. A three panel sequence with each panel connected with bi-directional arrows. Panel one refers to intervention strategies, panel two refers to implementation strategies, panel three refers to outcomes. Panel three is divided into three smaller panels that show implementation outcomes, service outcomes, and client outcomes.

This was a hybrid effectiveness-implementation feasibility study that sought to evaluate outcomes from AoP@Home programs for people with dementia and family carers and, in parallel, evaluate the implementation process. Figure 1 illustrates the project model, showing the intervention strategy (AoP@Home) and the implementation strategies as two separate domains, with outcomes evaluated across both.

Community aged care in Australia is subsidised by government funding whereby after assessment of level of need, packages of funding support^24^ or programs of services^25^ are assigned to older people to use towards services and care.^26^ This funding follows a consumer directed care model where people are able to choose which approved service providers to access with their assigned funding.

The current study was conducted within an existing community aged care service provider in Sydney, Australia. Among other services, the provider offers a range of community home care services via a team of care managers and trained aged care workers. In parallel, occupational therapists, exercise physiologists, physiotherapists, and artists make up their multidisciplinary allied health service. The artists (who encompass both AoP program managers and AoP artists) on the team have been providing group-based arts programs since 2015, and also undertook the recently published AoP@Home pilot study,^8^ but had not been routinely offering AoP@Home prior to the current study. The largely casual team of AoP artists are scheduled to work when AoP group classes or AoP@Home programs are assigned to them. When the AoP service was established, this causal employment of the AoP artists facilitated flexibility and sustainability of the service. Referrals to AoP@Home can be generated from within the community aged care provider (e.g. from care managers or the allied health team), or from the broader community and aged care sector (e.g. from general practitioners or other care providers).

**Figure 2.**
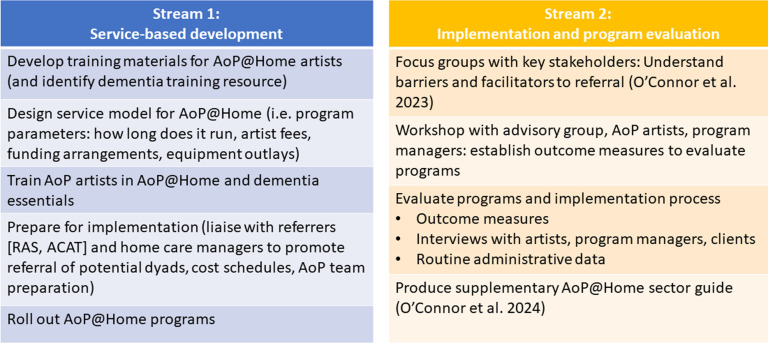
Complementary project activity streams to facilitate program roll-out and evaluation. *Notes*: ACAT – Aged Care Assessment Team; AoP@Home – Arts on Prescription at Home; RAS – Regional Assessment service; Stream 1: service-based development activities (undertaken by the AoP team); Stream 2: research-based implementation and program evaluation activities (undertaken by the research team). A two panel table with panel one listing service-based development activities, and panel two listing implementation and program evaluation activities.

This implementation study involved a synergistic process whereby two complementary streams of project activities were undertaken in parallel to achieve the project aims (Figure 2). Stream one involved service-based development activities (undertaken by the AoP team), and stream two involved the research-based implementation and program evaluation activities (undertaken by the research team). At the beginning of the project, an advisory group consisting of people with lived experience of dementia and or memory impairment and family carers (n = 4) was established to work in partnership with the research team to provide expert knowledge, perspective, and feedback throughout the project. Specifically, the advisory group were consulted on the education topics for AoP@Home artist training materials, focus group questions, AoP@Home sector guide, and either attended (or commented on) the workshop to determine program outcome measures (Figure 2).

### Participants

AoP@Home was delivered as part of routine care at the community aged care provider; participants were identified through this process, therefore, initial contact with potential participants occurred when a referral was received by the AoP team. All participants had to be at least 18 years of age. All dyads (person with dementia and informal carer) who enrolled in an AoP@Home program during the study period were invited to participate in the research evaluation. The other inclusion criterion for dyads was that people with dementia deemed not to have capacity to provide their own consent required a family carer or appropriate person responsible to consent and be willing to support that person’s participation in the research. Assessment of capacity was completed by the community aged care provider, which was informed by the referral information provided upon admission to the service. In the situation where a person was deemed not to have capacity to provide their own consent, proxy informed consent was provided by an appropriate person responsible who also supported the person’s research participation. In parallel, assent for participation was sought from the person with dementia.^27^

Artists who had completed training in AoP, AoP@Home, and Dementia Essentials (all delivered in house via the AoP team or the broader aged care organisation), and who were involved in delivery of AoP@Home during the study period were invited to participate. AoP program managers who were involved in coordinating the delivery of AoP@Home programs during the study period (e.g. receiving referrals, assigning artists, linking funding etc) were also invited to participate. Ethics approval was provided by the University of New South Wales Human Research Ethics Committee (HC210033) and all participants provided informed consent to participate.

### Intervention Strategy - AoP@Home

AoP@Home involves a professional artist visiting a client with dementia and their family carer(s) to engage them in participatory art in the home over eight weekly sessions. The art form used depends on client preference and interest, and can include visual arts (e.g. drawing, painting, sculpture, clay work), music (e.g. could involve singing or a range of musical instruments), creative movement and dance (e.g. using gentle body movement and dance to generate enjoyment, while also enhancing balance, confidence and flexibility), and dramatic arts (e.g. dramatic performance and storytelling). An eight-week program comprises three phases: (1) an introductory phase (weeks 1-2) that involves rapport building, identifying creative interests and planning the program; (2) an art-making phase (weeks 3-6) that involves experimenting and practicing with techniques and skills while engaging in art-making activities; and (3) a reflection and celebration phase (weeks 7-8) that involves reviewing, reflecting on, and celebrating the art-making journey.^8,28^

### Implementation Strategies

The implementation plan used by the service provider to support AoP@Home roll-out (Figure 1) was drawn from focus groups that were conducted with key stakeholders to explore the barriers/enablers around implementing AoP@Home. Prior to conducting the focus groups, the draft questions were reviewed by the project advisory group to ensure topics covered would be relevant and appropriate to people with dementia. Outcomes from the focus groups (which have been published previously;^19^) were reviewed within the context of the Consolidated Framework for Implementation Research (CFIR;^29^) and supplemented with the Expert Recommendations for Implementing Change (ERIC) strategy compilation.^30^ Specific strategies implemented by the service included:

*Promotion*: A new AoP@Home flyer was developed and distributed locally to referrers (i.e. care managers from the broader community aged care service provider) and potential clients who were receiving other care and services through the community aged care provider. Information on the government referral portal (My Aged Care) was updated to include specific information about AoP@Home and how to link with government funding mechanisms. The service provider appointed an AoP program manager to coordinate the AoP@Home service and act as a specific liaison to promote the program.

*Education*: As part of stream 1 activities (Figure 2), the AoP team developed an AoP@Home online training resource designed to be supplemental to the original AoP training^15^ and dementia essentials training (both delivered in-house by the aged care organisation). Prior to content development of the education modules for this training, the education topics that were to be included were provided to the advisory group to ensure the relevance of the artist education topics. In addition, the AoP program manager liaised with referrers, care managers and aged care workers to provide top-down and bottom-up education on AoP@Home. The community home care services team was requested to share the AoP@Home flyer with their clients.

*Adapting to context (practicalities)*: The AoP service-based development team designed a service model for AoP@Home that would fit within the current Australian government funding system for community aged care services. Additional considerations were program length, session length, artist fees and equipment costs, referral pathways, and logistics around staffing via a casual artist workforce that would incorporate sufficient spread over Sydney in addition to a range of art form specialities (Figure 2). The AoP@Home protocol that was drafted during this process and then implemented during this feasibility study was subsequently developed into a freely available AoP@Home sector guide.^28^ During development of the AoP@Home sector guide, the advisory group was invited to review and provide feedback to ensure content and themes within the guide were appropriate to people impacted by dementia.

*Sustainability*: An AoP hub (i.e. website landing page via the community aged care provider) is currently being developed to facilitate broad access to information about the range of AoP programs, including AoP@Home.

### Evaluation

#### AoP@Home program outcomes for the person with dementia and family carer

In July 2021 a workshop was facilitated by the researchers (CMCOC, CP), with representation from the advisory group (n = 1 attended, n = 2 consulted after the workshop), AoP artists (n = 2), and program managers (n = 2), to determine how best to evaluate the outcomes for both the person with dementia and the family carer(s) participating in an AoP@Home program. Involving members of the lived experience advisory group in this workshop was vital to optimise the study design and ensure that outcomes were relevant.^31^ Participants in the workshop were clear that the outcome measures needed to be practical and feasible within a busy clinical context (i.e. brief) and capture key outcomes specific to potential benefits of engaging in an AoP@Home program. This aligns with previous research highlighting that Patient-Reported Outcome Measures should capture outcomes that are valued by the clients and facilitate tailored care planning and ongoing service development.^32,33^ The final agreed upon measures included health and wellbeing, and personal goal attainment.

Health and wellbeing: To measure health and wellbeing, the person with dementia was asked to mark the response that best described how they felt on the day for two general health and wellbeing questions: 1) ‘Overall, how would you rate your health?’ and 2) ‘Overall, how would you rate your well-being?’ The questions used a 5-point picture-based Likert scale (from ‘very poor’ to ‘very good’), and were adapted from the Australian Dementia Network patient survey^34,35^; the health and wellbeing items were originally drawn from the Bath Assessment of Subjective Quality of Life in Dementia (BASQID)^36^ and WHOQoL.^37^ The carer was asked to complete the Warwick-Edinburgh Mental Wellbeing Scale (WEMWBS;^38^). The suggested cut-off score of 42 for the WEMWBS was used to interpret scores, with lower scores indicative of poorer wellbeing. Both of these outcomes were collected pre- (at the beginning of the first art session, prior to any art activities) and post- (at the conclusion of the final art session) program by the AoP team.

Goal attainment: Goal attainment for both the person with dementia and carer was rated using a Goal Attainment Scaling – Light methodology previously developed for the evaluation of reablement programs for people with dementia^39,40^ and adapted specifically for use with arts programs for the current study.^41^ During implementation, artists identified a need to focus on immediate rapport building and creating a space where the aim was around participation in the art. Therefore, rather than using Goal Attainment Scaling as originally intended (i.e. in a formal, structured manner), the artist explored goals less formally through discussion with clients. After this initial session with the client, goal setting was then facilitated by the AoP manager in consultation with the artist, using the artist’s detailed notes. As per Goal Attainment Scaling methodology, at the end of the program, these goals were revisited with clients, and the clients were asked whether they felt like their goals had been achieved, and if, so, whether it was achieved ‘as expected’, ‘a little more’, or ‘a lot more’ than expected. Or if they believed their goal was not achieved, whether it was ‘partially achieved’, ‘no change’, or ‘got worse’.

Experience: The workshop did not cover experience measures, however, they are an important method of collecting information about client perspectives and experiences to facilitate quality monitoring and service improvement.^42^ At the end of the program, carers were asked to respond to three experience measures: 1) ‘I feel that my interactions with X have improved because of AoP@Home’ (5-point scale from ‘strongly disagree’ to ‘strongly agree); 2) ‘my overall experience of the AoP@Home program was: (5-point scale from ‘very poor’ to ‘very good’); and 3) ‘how likely would you be to recommend AoP@Home for others living with dementia?’ (10-point scale from ‘not likely at all’ to ‘extremely likely’). For this outcome, carers were provided with an envelope in which to seal their responses so that only the research team would see the outcome and the artist themselves would not.

### Implementation and Service outcomes

Implementation outcomes were evaluated according to feasibility, fidelity, acceptability, uptake, and costs (^43^; Figure 1). Routine administrative data collected by the service was used to extract this information, in addition to semi-structured interviews (facilitated by the researchers CMCOC, CP) conducted with clients, artists, and AoP program managers involved in AoP@Home programs. Interviews with clients included both members of the dyad and were conducted with all dyads on completion of their AoP@Home program in their home. Interviews with AoP@Home artists were conducted on completion of their first AoP@Home program during the study period, and interviews with AoP program managers were conducted after the final AoP@Home program during the study period was initiated; these staff interviews were either conducted face-to-face or via video-conference. Interview questions were designed to explore experiences around AoP@Home implementation and were based on questions used in the original pilot of AoP@Home.^8^ Specific questions for each stakeholder group were used to explore a range of implementation experiences, for example, participating in, delivering, and coordinating AoP@Home programs.^44^

For feasibility, artist training, artist recruitment/retention, and program participation rates were evaluated. For fidelity, artist notes were reviewed to explore adherence to the program protocol, client involvement, dose or amount of program delivered, and quality of program delivery. Artists completed an in-house reflective diary sheet at the conclusion of each session to capture what was covered during the session and artist reflections on what worked well and any challenges experienced during the session.^28^ Acceptability was evaluated via the experience outcome measures, and interviews with clients and staff, with consideration of program content, satisfaction, practicality, and suitability. Uptake was evaluated by reviewing the number of referrals received and the number of programs delivered, and costs were evaluated by reviewing funding used, artist fees, and equipment purchased.

The service outcomes were based on the Institute of Medicine (IOM) Standards of Care (^45^; Figure 1). Administrative data were used to evaluate efficiency (monitoring costs of materials used per program), safety (monitoring incident reports), timeliness (referrals vs programs delivered), and effectiveness and equity (monitoring triage process for referrals). Client centredness was evaluated using artist notes and client reported outcome and experience measures (i.e. goal attainment, health and wellbeing, program experience ratings).

### Data Analysis

Quantitative health and wellbeing data were analysed descriptively in Excel, with individual pre-post scores presented due to the small sample size. Goals were reviewed and categorised according to an ‘AoP client goal list’ developed in-house by the AoP team through their experiences delivering arts programs with older clients and clients with dementia.^41^ The list outlines five goal domains that commonly feature in discussions with clients at the beginning of their AoP program: skills acquisition, skills development, personal growth and achievement, health and wellbeing, and socialisation and social support.

Administrative data and artist notes were analysed qualitatively in Excel to identify key themes relating to specific implementation and service outcomes. Interview recordings were transcribed verbatim by an external transcription service. Transcripts were analysed by the first author using a naturalistic paradigm involving a combination of inductive (conventional) and deductive (directed) qualitative content analysis.^46^ That is, interview questions were initially used to address the research aims, then the data were reviewed more broadly to explore whether any further themes were apparent.^47^ Interviews across three stakeholder groups (AoP program managers, artists, AoP@Home clients) facilitated data triangulation for a comprehensive evaluation of the programs and implementation process.^48^ Data were initially coded, and then grouped to form broader themes.^49^ Verbatim quotes were extracted to illustrate themes with the following coding structure: participant group (AoP program manager – Mx, artist – A, person with dementia – D, family carer – C), participant number (e.g. Mx1-2, A1-3, D1-4, C1-4).

## Results

**Figure 3.**
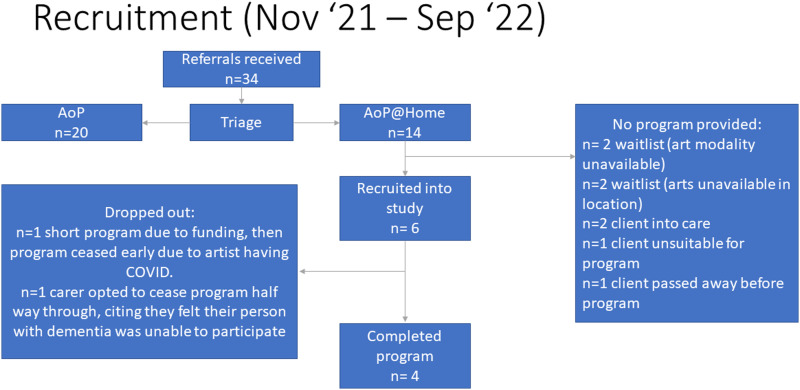
Recruitment flow-chart during the study period. *Notes*: AoP – Arts on Prescription (group-based programs); AoP@Home – Arts on Prescription at Home. Multi-level flow chart outlining the number of referrals received, the number of participants recruited, number of participants who completed the program, number of participants who did not receive a program and reasons why, and the number of participants who dropped out and reasons why.

During the study recruitment period (Nov 2021 – Sept 2022), the AoP team received 34 referrals for AoP. The AoP program managers then triaged these to determine which clients might be suitable for the AoP group-based programs (i.e. would be able to attend the location of the arts classes, and effectively participate and engage in the arts process in a group-based setting; n = 20), and which clients might be more suited to AoP@Home (i.e. may have higher level support needs due to their stage of dementia, which might make attending and effectively participating in a group-based setting more challenging; n = 14). By the end of the recruitment period, eight of the 14 clients triaged for AoP@Home had not received a program (reasons outlined in Figure 3), and six clients had been recruited into the study and initiated an AoP@Home program, however two of these did not complete their programs and dropped out of the study (reasons outlined in Figure 3). Four clients who did not receive a program during the study period were on a waitlist; two of these clients had requested music as their art modality, which could not be fulfilled during the study period due to limited artist availability; the other two were geographically located beyond the reach of the arts team during the study period. More detail around the triage process, and the factors considered in triaging each referral, is provided below under the section ‘*Effectiveness and equity’*. For a more in-depth exploration around stakeholder perspectives and experiences from the implementation process, see O’Connor et al.^44^

Table 1 provides an overview of the demographic information of participants. In brief, the majority of participants with dementia were female (3/4), with ages ranging from 81-90 years. Three programs were visual arts, and the fourth program was music and dance. All carers were female, lived with the person with dementia, and the majority were the daughter (3/4) of the person with dementia, with one wife. While AoP@Home was designed to be a dyadic program where both members of the dyad are active participants in the art making, only one carer in this cohort chose this option, with the other three preferring to use the session time as respite.

**Table 1.** Participant Baseline Demographic Details.

	1	2	3	4
Person with dementia				
**Age** (years)	88	81	90	90
**Sex**	M	F	F	F
Dementia information as per referral notes	Lewy body dementia	Moderate dementia	Moderate dementia	Dementia
**Referral reason/aims for the program stated in referral**	Cognitive stimulation	Physical activity, mental health, social connections, cognitive stimulation, new interests	Social connections, cognitive stimulation, new interests	Cognitive stimulation
**Client stated goals**	Skills development; skills acquisition; personal growth and achievement	Personal growth and achievement; socialisation and social support	Skills acquisition; health and wellbeing; socialisation and social support	Skills acquisition; skills development; socialisation and social support
**Referral source**	Care manager	Care manager	Exercise physiologist	Care manager
**Program funding**	Commonwealth HCP	Commonwealth HCP	Commonwealth HCP	Commonwealth HCP
**Program medium**	Visual arts	Visual arts	Music and dance	Visual arts
Family carer				
**Age** (years)	88	56	57	65
**Sex**	F	F	F	F
**Relationship to person with dementia**	Wife	Daughter	Daughter	Daughter
**Lives with person**	Yes	Yes	Yes	Yes
**Actively participated in AoP@Home program**	No	Yes	No	No
**Carer stated goals**	For self: Health and wellbeing; socialisation and social support	For self: Health and wellbeing	For mother: Skills acquisition; personal growth and achievement; health and wellbeing; socialisation and social support	For mother: Health and wellbeing; socialisation and social support. For self: Skills acquisition; health and wellbeing

### AoP@Home program outcomes for the person with dementia and family carer

**Figure 4.**
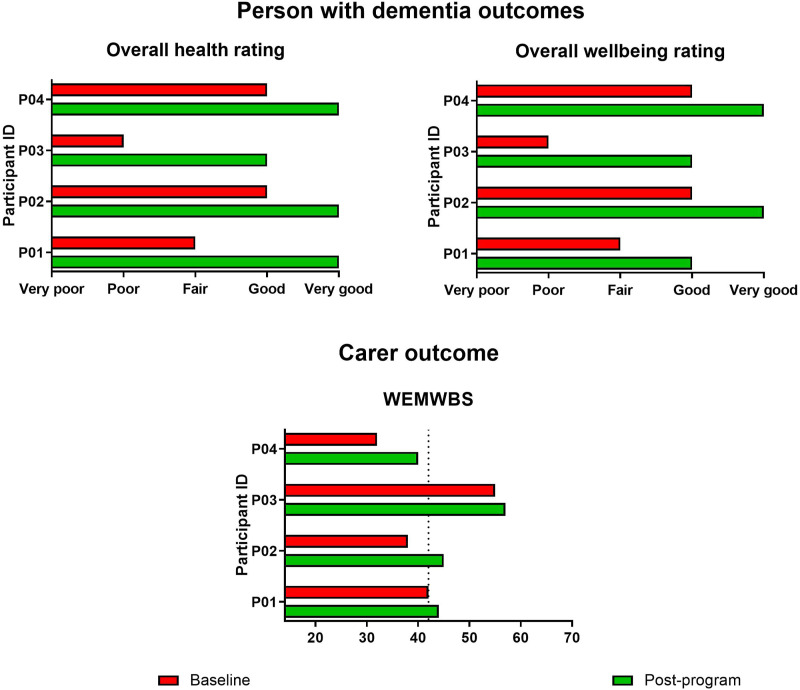
Health and wellbeing outcomes for people with dementia and family carers. *Notes*: WEMWBS – Warwick Edinburg Mental Wellbeing Scale; the dotted line indicates the cut-off for wellbeing. Three separate graphs, with each showing baseline and post-program outcomes for the program participants. The first graph shows overall health rating scores for people with dementia. The second graph shows overall wellbeing scores for people with dementia. The third graph shows wellbeing scores for family carers.

*Person with dementia wellbeing*: All participants living with dementia were able to engage with the outcome measure, and reported improvements in their overall health and wellbeing from baseline to post-program (Figure 4).

*Carer wellbeing*: Carers were all able to self-complete the WEMWBS. Scores on the WEMWBS improved for all carers from baseline to post-program (Figure 4). At baseline, three carers scored ≤42, suggesting they were experiencing lower wellbeing; post-program, two of these carers had improved scores so that they were scoring above this cut-off.^50^ Two carers had scores that improved by ≥ 7 points, which is well above the 3-point change suggested to indicate clinically significant individual change.^51^

*Goals:* Every person with dementia was able to discuss goals with the artist. They had goals associated with skills acquisition (e.g. experiment and learn with some new materials to me [D1 – from artist notes]), three identified goals for socialisation and social support (e.g. *“Better to have it ‘in company’”* [D2], two for personal growth and achievement (e.g. *“Do something productive, releasing energy because you achieve something.”* [D2]), and two for health and wellbeing (e.g. wants to feel stronger, easier to move [D3 – from artist notes]). Two carers identified goals they hoped their person with dementia would achieve, both citing health and wellbeing, and socialisation and social support. Three carers reported goals for themselves, with all three citing health and wellbeing, one identifying skills acquisition, and one for socialisation and support. At the end of the program, all people with dementia felt like they had achieved their goals ‘a lot more than expected’, with one participant exclaiming *“now I’m an artist!”* [D4]. Carers who had identified goals for their person with dementia also felt like their goals had been achieved ‘a lot more than expected’. Of the carers who identified goals for themselves, one carer felt like their health and wellbeing goal had been achieved ‘a little more than expected’, and all the other goals were achieved a ‘lot more than expected’.

### Implementation and service outcomes for AoP@Home programs

*Feasibility and Timeliness:* During the study period, nine AoP artists completed the AoP@Home training and the additional in-house dementia training; four of these artists delivered AoP@Home programs, two artists left the organisation, and the remaining three were not matched with an appropriate client in their art modality and location. As per the flow-chart in Figure 3, limitations around service reach due to artist availability (related to either geography or specific art modality) impacted on how many referrals were able to be converted into delivered programs.

AoP program managers reported that the specific AoP@Home training on top of the foundational AoP training and dementia training was vital as it was *‘tailored directly to what the artists were going to be doing’* (Mx1) and that *‘it definitely enabled people to feel ready to go into the home’* (Mx2). Artists agreed the training *‘was good’* (A2) and *‘that [it] definitely helped, that training session and it complemented my previous dementia training’* (A3). AoP program managers also commented on the training creating a unifying team environment: *‘I think that the key thing about training … in terms of it being ongoing or feeding into the service more widely, is that it’s that opportunity for them [the artists] to connect with each other ... To be able to share experiences and also to be able to problem solve together as a team, that they’re not just relying upon the training or the one coordinator to give that, but that it’s more dynamic, they give it to each other’* (Mx2). Overall, this feedback indicates the importance of tailored education, ongoing support, and a collaborative work environment to support program implementation.

Of the two participant dyads who dropped out of their AoP@Home program, one had begun a planned program of four sessions by adding an AoP@Home component to a defined, government funded restorative care program. However, after two weeks of their AoP@Home program, the program was terminated due to the artist contracting COVID-19. The second dyad who dropped out participated in four sessions before the family carer decided to pull out of the program citing a belief that the person with dementia was too impaired to participate in the program.

Regarding timeliness, for the four programs that were delivered during the study period, two programs were initiated in less than a month since referral, and two were initiated after a one-two month wait. Four clients remain on the waiting list due to unavailability of their preferred art form in their area, or unavailability of an artist in their area (Figure 3).

*Fidelity:* Of the four participants who completed their AoP@Home program, the majority (3/4) completed eight sessions with the artist, as suggested in the protocol.^28^ The fourth participant received their program over six sessions due to pragmatic reasons with their program falling over the end of year period; this was planned a priori. The average face-to-face session length was 83.8 minutes (range 60 – 120 minutes).

All artists completed the reflective diary sheet after each AoP@Home session. These notes indicated that the artists established an ongoing, personal relationship with both the person with dementia and their family carers, whether the carer was actively participating in the program or not. As per the AoP@Home protocol within the sector guide,^28^ artists reviewed work with their clients from the previous week, planned the ongoing program together, engaged in art-making, and built on techniques and skills. At the end of each visual arts program, each artist facilitated a celebration of the client’s art making journey and their work. The celebrations involved a range of approaches including a mini exhibition, a folder portfolio photo record, and framing the client’s major art work. Notes reflected an overall high level of engagement with the program from each of the clients with dementia. Where there were challenges around engagement, artists were able to implement strategies to help the client re-engage, such as using familiar music, changing activities when needed, and using familiar items as a prompt.

**Figure 5.**
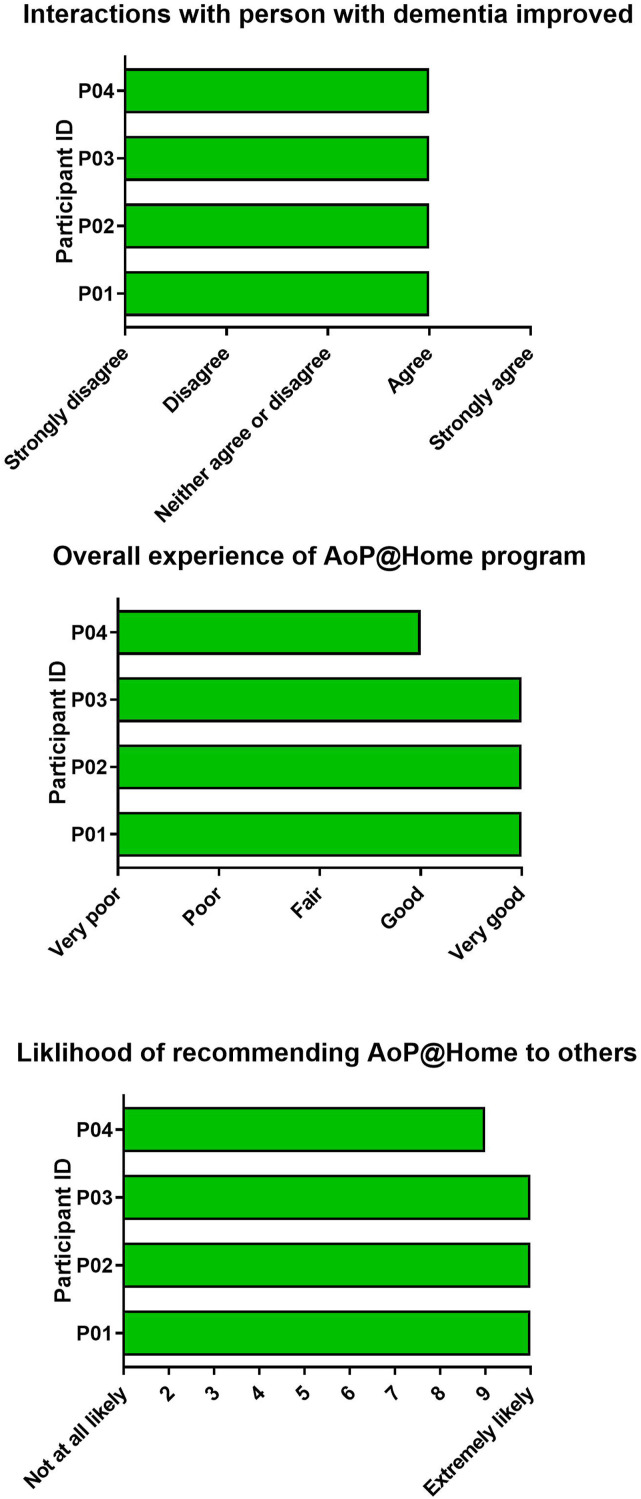
Experience measures completed by family carers post-program. *Notes*: Three separate graphs showing outcomes from experience measures. The first graph shows ratings for whether carers felt that their interactions with the person with dementia improved. The second graph shows ratings for the overall experience of the AoP at Home program. The third graph shows ratings for the likelihood of recommending AoP at Home to others.

*Acceptability and Client centredness:* On the experience measures, all carers agreed that their interactions with the person with dementia had improved because of AoP@Home. The majority of carers reported that their overall experience of AoP@Home was very good, and the majority of carers reported that they would be extremely likely to recommend AoP@Home for others living with dementia (Figure 5).

AoP program managers and AoP artists described their experiences coordinating or delivering AoP@Home programs. Managers shared their views on the positive impact of the program for *‘not just the person with dementia but the people who are living with the person and caring for them as well’* (Mx1). Artists shared similar views around the importance of AoP@Home to support people with dementia who may be unable to attend group-based services. Artist shared that *‘this program is so essential and I hope it’s going to take off and run more’* (A1), and that ‘*it’s a really important service [for] people … who because of their dementia they’re not able to go into the world and meet new people with as much ease as they could in the past, so bringing [a] fulfilling and engaging and interesting experience to them I think is just so important because it felt like a day out to [my client with dementia]. She loved meeting a new person and having a new experience even though she was in her same living room that she’s always in, it felt new’* (A3).

The process of coordinating the service led AoP program managers to understand *‘how much work it does take to be able to implement something new like this and what the demands are for implementation’* (Mx1). While there were many positives, challenges around implementing AoP@Home were also discussed, including limitations around the small service size and generating referrals: *‘it’s been pretty frustrating to have it not be able to move at the pace that I think that it has needed to, it’s needed a lot more. Not being able to offer that many [programs] has meant that the momentum hasn’t been shared and therefore we haven’t got more people to kind of connect’* (Mx2).

Clients reported that the AoP@Home programs *‘more than met my expectations’* (C2), with benefits reported for both the person with dementia and the family carer. People with dementia reported *‘I certainly enjoyed it’* (D1), and that they felt a sense of purpose and achievement through participating in the program.

*Gives you a bit of – oh, what's the word – a bit of a push to know that what you're doing is worthwhile and some may appreciate it. *(D2)

*It makes you feel proud, what you can do ... It makes you feel good and “look what I did”. How much is it worth? ... Where are we going to put it on the wall? ... Well, for a first time, I reckon it’s a good job ... If I do say so myself ... I’m a hidden artist.* (D4)

Carers agreed that the person with dementia enjoyed participating in the program, listing positive aspects such as progression in art skills, social engagement, boosted mood, and sustained enthusiasm beyond the end of the program session. Carers particularly emphasised the level of engagement that was generated in the person with dementia during sessions, and the person’s sense of achievement and boost of confidence they received.

*… just the continuing benefit of just her realising how good she is at art, sort of that confidence to say gosh, did I do that? Oh, isn't it good? Yeah. Well I'm pretty good aren't I? Yeah, just that boost, yeah.* (C2)

*… my mum was completely engaged for the whole time … you know her dementia is pretty advanced, so I actually think music really transcends all of that stuff you know.* (C3)

The majority of carers (3/4) chose to spend the session time as respite, stating *‘having someone come in and take the reins for an hour is really what’s so very valuable to us’* (C3). Despite this, carers reported benefits from observing the person with dementia engaged in the program, such as developing understanding around the person’s abilities, and gaining ideas for future engagement with their person beyond the program.

*It also gave me the opportunity to see what she can actually do ..*. *It was totally unknown to me what she could do … it was just beautiful to see her engaged in something. Concentrating on something different. Listening to [the artist]. Being guided by [the artist]* (C4)

*… just an absolute joy to see my mother having fun with somebody you know – like it’s fantastic. It’s amazing actually* (C3)

The carer who did participate actively in the AoP@Home program agreed that *‘it was just good to be involved in something with her and not have to be instructive’* (C2), however reported specific benefits from having a shared engagement by being able to do art alongside her mother with dementia: *‘it was good to work on a project with mum, so that was lovely to share that, rather than being the watcher, to get involved and just enjoy the journey together I suppose’* (C2).

*Uptake:* During the study period, n = 14 AoP@Home referrals were received and n = 4 programs were delivered in full. The number of delivered programs compared to program referrals was impacted by a number of factors including COVID-19 restrictions, completion of artist training, and a casual artist workforce leading to challenges in linking up referrals with appropriate art modalities within geographical regions of Sydney. Regarding the two participants who initiated an AoP program and subsequently dropped out:

1. One participant (on a time limited funding stream) ceased their program early (after two sessions) when their artist contracted COVID-19. Their carer felt it was not appropriate to complete the post-program outcome measures after such a brief time.
2. One participant ceased their program early because their carer felt the participant (person with dementia) was unable to appropriately engage in the program.

*Costs and Efficiency:* All completed programs were delivered using government ‘Home Care package’ funding.^24^ Programs were costed at AU$230 per session, so that an eight-week program cost AU$1840. Art materials were purchased in bulk by the AoP team and used across the AoP programming, which included AoP@Home. Each artist was able to select from this pool of supplies when planning their AoP@Home program. An allowance of AU$120-150 worth of art supplies was in-built into the costs for an eight week program. While one carer shared that *‘whilst this is a great idea, I thought the cost of it was quite expensive’* (C4), overall, clients reported that the cost of the program was *‘well worth it, absolutely well worth it’* (C2), and that the government funded packages made a big difference to ensuring the program was accessible for them: *‘Well, it doesn’t cost us anything because [de-identified] has got a package from the government. So it doesn’t cost us’* (C1).

*Safety:* No incident reports were lodged throughout the study period for any of the AoP@Home programs.

*Effectiveness and equity:* When a referral for AoP came through to the team, an initial triage was undertaken by the AoP program managers where anyone with a diagnosis of dementia was considered for AoP@Home. This triage process considered a range of factors to determine which of these referrals might be appropriate for AoP@Home, including: the carer, the person with dementia, and the artist. Reported carer factors were around hesitation committing to something they feel might add further stress on them and deciding that they no longer want to participate in the program. Factors associated with the person with dementia related to whether the person has complex behaviour that might need consideration prior to an artist delivering a program in the person’s home. Artist factors were around availability of artists (i.e geography or art modality to match client preferences) or suitability of an artist for a particular client (e.g. client preference for a female artist).

## Discussion

This hybrid-effectiveness feasibility study was the first to evaluate implementation of AoP@Home for people living with dementia and their family carers within a real-world community aged care context, and has demonstrated the feasibility of using existing funding mechanisms to support program roll-out. As previously recommended,^19,52^ the AoP team used a range of implementation strategies to support program roll-out, and despite some identified challenges around implementation, artists and program managers believed AoP@Home is an important program for people living with dementia and their families. This was also the first evaluation of AoP@Home program outcomes when delivered within a real-world service context. People living with dementia and family carers in this study benefited from engagement with the program in terms of health and wellbeing and positive experiences. For a more in-depth exploration of the experiences of key stakeholders involved in this implementation study, see O’Connor et al.^44^

The decision to measure health and wellbeing as the primary clinical outcomes for AoP@Home programs over other often generically measured domains in dementia, such as cognition, aligns with recent research about likely program impacts.^53^ To date there is no gold standard approach to measurement of health and wellbeing for people with dementia. Research has highlighted differences between self and proxy ratings for people with dementia,^54,55^ and there is no consensus around which approach is most accurate.^56,57^ Large trial studies often use multiple measures in parallel to address this potential issue,^57,58^ however, in the current pragmatic implementation study, the evaluation approach was directly informed by the workshop participants (advisory group, AoP artists, and AoP program managers). While the self-report outcomes used to evaluate health and wellbeing for people with dementia are an unvalidated measure, these were adapted from the patient survey used for the national Australian Dementia Network Registry and is therefore a nationally recognised approach to gather self-report data from people living with dementia.^34,35^ Preliminary outcomes from the current study suggest that the selected outcomes were appropriate for evaluating AoP@Home programs in this setting, with scores on health and wellbeing improving for all participants with dementia after having engaged in the AoP@Home program. Having access to individualised interventions that promote engagement in meaningful activity and support relationships, such as AoP@Home, has been identified as an important component to post-diagnostic support that relates to psychological and emotional wellbeing.^59^ Indeed, participants with dementia reported feeling a sense of purpose and achievement from participating in AoP@Home, which reflects outcomes from the previous AoP@Home pilot study.^8^

Family carers similarly reported benefits they observed in their person with dementia, such as improved confidence and the level of engagement their person demonstrated during AoP@Home sessions. Dyadic interventions such as AoP@Home have been shown to benefit both the person with dementia and their family carers.^60^ Interestingly, in the current study, only one of the carers opted to participate actively in the AoP@Home program, while the other three opted to use the time primarily as respite. Despite this, in addition to benefits in their person with dementia, carer wellbeing scores improved for all participants in this study. In-home respite has been shown to benefit carers of people with dementia,^61^ yet there are a range of barriers to carers accessing respite services, including (but not limited to) a fragmented care system, lack of availability, and guilt.^62,63^ Tailored services are needed to support carers in using respite,^63^ therefore, it is possible that for the carers who opted to use the AoP@Home session time as respite, the program provided an accessible avenue for accessing much needed respite. Carer perception of mutual benefit for both the person with dementia and the carer from a service has been identified as an important factor in positive experiences of respite,^64^ and carers in the current study reported benefits from observing AoP@Home engagement in their person with dementia.

The concept of respite also extended to the carer who did actively participate in the program in that they appreciated being able to be engaged in the AoP@Home sessions and not be ‘caring’ during that time, which aligns with findings from the original pilot study for AoP@Home.^8^ Additionally, this carer reported enjoyment from the experience of shared engagement in art making with her mother with dementia. Carer-reported benefits from engaging in shared meaningful activities with their person with dementia have been previously reported.^8,65–67^ Finally, carers who were both actively engaged in the AoP@Home program or who used the time primarily as respite identified benefits from AoP@Home through developing a greater understanding around their person with dementia’s abilities and gaining ideas for future engagement with their person beyond the program, which reflects previous dyadic studies that involve active engagement in individualised activities.^66,67^

For the AoP team, challenges were identified around the implementation of AoP@Home. As with the broader aged care sector,^68^ COVID-19 restrictions had an impact on referrals and study recruitment. In parallel, the requirement for the service team to develop the AoP@Home artist training and train the artist team prior to program roll out also contributed to delays. This need for adequate time for development and planning prior to implementation has been previously highlighted.^69^ Despite the associated added delays, the artist training in AoP@Home and working with people with dementia was seen as vital to support confidence and skills in service delivery with this specialised client cohort and in creating a community of practice amongst the artist team. Indeed, the importance of developing skills in working with people with dementia in order to effectively deliver interventions to this cohort, and the need for formal training to address this need has been previously identified.^70^ Further, this process contributes to building a sense of ownership and shared perspectives around the value of AoP@Home, which ultimately contributes to successful implementation of programs.^70^ In the current study, artist notes indicated that artists were able to effectively apply skills they had learned in their training to support engagement with their client with dementia within the AoP@Home sessions.

An in-depth discussion around delivering AoP@Home within the home setting is included in a supplementary paper.^44^ In brief, clients, artists, and program managers all agreed that being able to deliver programs within the home was a benefit. In parallel, artists discussed some of the practicalities around delivering a program in the home, with the need to consider specific factors such as the physical environment (e.g. living room spaces), being adequately prepared and bringing all required equipment, and navigating potentially complex family dynamics.^44^ This concept of working with families within the home has also been raised in broader dementia care research,^71^ and the artists in the present study commented on the potential to include family where possible and observe family dynamics to gain insight into their client and their environment.^44^

AoP program managers discussed challenges around linking referrals for AoP@Home with appropriate art modalities within geographical regions of Sydney. Interestingly, this issue was raised as a potential barrier in the implementation preparation phase of this study.^19^ The specialised nature of each arts modality (e.g. visual arts, movement and dance, music), means that each artist trained in AoP@Home is only a specialist in their specific modality. Therefore, if a prospective client was referred specifically for a music program and the music artist that was available lived on the other side of the city, that client would go onto a wait-list until the AoP service was able to bring on a music artist within the client’s geographical area. The small size of the program meant that individual disciplines were represented by only one or two artists, thus we suffered from a lack of ‘economies of scale’. A further limitation to this service limitation was that just over 20% (2/9) of the artists initially trained in AoP@Home left the service during the study period. While this is less than previously reported drop-outs from trained therapists in a recent Australian implementation trial,^69^ it still impacted on the ability for the service to link referrals received with appropriate AoP@Home artists. It is possible that the largely casual basis of artist employment within this service at the time of the study, contributed to increased likelihood of artists leaving the role for a more permanent position.^72^ More stable funding sources are now available, and the service is able to offer permanent positions for artists which may improve artist retention. The involvement of artists within the health and aged care field is only in its infancy in Australia. It is not yet a career that artists look to, and all artists need additional training before moving into this field. However, we have found a great deal of interest from artists to be involved in the AoP program. AoP@home is an “artist intensive” program, but essential to meet the needs of certain people with dementia. Group-based programs allow one artist to provide a program for multiple participants, and is therefore more effective in terms of artist-participant ratio. But such a program will not suit, or meet the needs of everyone. Finally, the occurrence of COVID-19 complicated service delivery, as it did in all health and community service settings. An interesting future direction of this work will be to explore the potential of delivering AoP@Home via telehealth. This would overcome barriers experienced in the present study such as COVID-19 and geographical limitations, and would enhance accessibility to the program beyond Sydney.

A positive outcome from the current study was around fidelity of program delivery according to the AoP@Home protocol within the sector guide.^28^ Implementing an intervention as close as intended to the protocol maximises the likelihood of positive program outcomes.^73^ In parallel, it is recognised that modifications are sometimes necessary to ‘fit’ an intervention within a real-world context.^74^ When working with people with dementia, it is especially important to be flexible in approach to accommodate any unpredictability that may occur during a planned session.^75^ This flexibility in approach was illustrated by the artists in the current study through the use of strategies to support the differing needs of each of the clients and promote engagement with the program.^44^ Within the current study, flexibility around program length was applied to align with available government funding structures. For example, one client program was planned by adding AoP@Home onto their already initiated government funded restorative care program which required coordination with the broader allied health team, and resulted in reduced planned program length. This need to determine how to leverage funding models to facilitate accessibility to programs has been highlighted from a recent Australian community aged care implementation study.^70^ Importantly, the cost for delivering AoP@Home programs was favourably comparable to similar in-home programs involving art or music therapists. Specifically, an art or music therapist costed at the average AoP@Home session length of 83.8 minutes would cost AU$270, which is AU$40 more than each AoP@Home session.^76^

This study has several limitations that need to be considered. The sample was smaller than originally intended, largely due to COVID-19-related delays; it is therefore important to note that the study participants are not representative of all people with dementia. While the outcomes presented are representative of these study participants only,^77^ the findings provide important insights into areas for further investigation. Beyond COVID however, the methodological challenges in arts and dementia evaluation and research have been recognised.^78^ In the present study, collection of health and wellbeing data at the beginning of the first session (prior to any art activity) and at the end of the last art session (after art celebration) may mean that for the person with dementia, these results reflected what happened in the moments prior to assessment, rather than representing a cumulative rating of the program as a whole at baseline vs post-program. Despite this limitation, providing feasibility data around AoP@Home within a real-world context is vital for supporting future large-scale implementation studies and to maximise access to these services for people living with dementia.^79^ An additional consideration is that artists themselves collected the outcome measures during their service delivery. While this may have introduced bias through lack of blinding and potential for clients to amend responses in the presence of the artist, as a real-world study, a practical, pragmatic approach to ongoing service monitoring was undertaken. This measurement process has also been used successfully in previous implementation studies in this population.^69^ Finally, the outcomes of this study would be enhanced by inclusion of a greater qualitative exploration around this implementation process, with specific input from the participants with dementia, however, this level of detail was beyond the scope of this feasibility study. To address this limitation, there is a supplementary manuscript that provides an in-depth qualitative exploration of the AoP@Home implementation process with key stakeholders including people with dementia and their family carer (i.e. both members of the dyad), AoP@Home artists, and AoP program managers.^44^

## Conclusions

This was the first evaluation of AoP@Home implementation into a real-world community aged care service context. AoP@Home was well-received by people with dementia and their family carers, with both members of the dyad experiencing benefits from the program. While practical challenges around COVID and appropriately matching artists with referrals were identified, successful programs with high fidelity to the AoP@Home protocol within the sector guide were able to be delivered using government funding models. These outcomes suggest that it is possible to deliver participatory arts programs for people living with dementia in the community, in their home, using sustainable and available funding models. Programs such as AoP@Home are a valuable tool for people living with dementia and their family carers, and should be made more accessible alongside broader allied health and care services.
